# When There is No Guidance From the Guidelines: Renal Transplantation in Recipients With Class III Obesity

**DOI:** 10.3389/ti.2023.11428

**Published:** 2023-09-15

**Authors:** Hannah Gillespie, Stephen O’Neill, Rebecca M. K. Curtis, Chris Callaghan, Aisling E. Courtney

**Affiliations:** ^1^ Regional Nephrology and Transplant Unit, Belfast City Hospital, Belfast, United Kingdom; ^2^ Statistics and Clinical Research, NHS Blood and Transplant, Watford, United Kingdom; ^3^ Department of Nephrology and Transplantation, Guy’s Hospital, London, United Kingdom

**Keywords:** end-stage renal disease, graft function, guidelines, kidney transplantation, transplant assessment

## Abstract

Whilst renal transplantation is the optimal treatment for many patients with end-stage kidney disease, the latest international guidelines are unable to make recommendations for the management of patients with end-stage kidney stage kidney disease and Class III Obesity (BMI ≥40 kg/m^2^). Data on all adult patients receiving a kidney-only-transplant in the UK between 2015–2021 were analysed from a prospectively collected database and interrogated across a range of parameters. We then analysed in detail the outcomes of patients transplanted at the highest-volume unit. There were 22,845 renal transplants in the study time-period; just 44 (0.2%) were performed in recipients with a BMI ≥40 kg/m^2^. Most transplant centres did not transplant any patients in this category. In the centre with the highest volume, there were 21 transplants (9 living donor) performed in 20 individuals (13 male, median age 46 years). One-year patient and death-censored graft survival was 95% and 85%. Successful transplantation is possible in patients with BMI ≥40 kg/m^2^ but carries additional risk. Obesity should not be the sole factor considered when deciding on transplant suitability. Restricting transplantation to a small number of high-volume centres in each country should be considered to optimize outcomes.

## Graphical Abstract

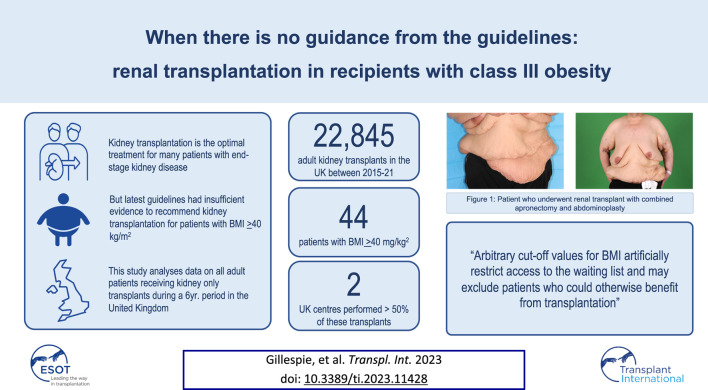

## Introduction

Globally, the prevalence of obesity has tripled since 1975, with a current estimate that over 650 million adults across the world are obese [1]. The rising prevalence of obesity in the general population is mirrored in patients with end-stage kidney disease (ESKD). This trend poses challenges to nephrologists and transplant surgeons alike [2]. Obesity can be a causative or contributing agent to the development of ESKD, may accelerate the progression to renal failure [3], and limit management options or efficacy.

Traditionally the metric used to categorise obesity is body mass index (BMI). It is easy to measure using routinely collected health data, and so has become a useful tool to correlate weight with adverse health outcomes at a population level [4]. Although imperfect, it remains a commonly used, easily measured and a practically useful measurement [5]. Obesity is defined as a BMI ≥30 kg/m^2^ and can be subdivided into classes I (BMI 30–34.9 kg/m^2^), II (BMI 35–39.9 kg/m^2^), and III (≥40 kg/m^2^).

Kidney transplantation is the “gold-standard” form of renal-replacement therapy, offering patients both improved quantity and quality of life compared to maintenance dialysis therapy [6]. Additionally, it is more cost effective [7, 8]. Obesity, however, confers additional risks to patients undergoing transplant. First, the hazards associated with general anaesthesia are magnified [9–12]. Second, there are greater technical challenges specific to transplant surgery including increased difficulty of vascular anastomoses, increased blood loss, potential for delayed graft function, and wound complications [13, 14]. And third, there is a higher likelihood of adverse outcomes related to long-term immunosuppression following transplantation, such as hypertension and diabetes mellitus [14–16].

Despite the rising prevalence of obesity within the ESKD population, only a small percentage are listed for transplantation [17]. Many guidelines exist to assist clinicians in assessing patients’ suitability for renal transplantation. The European Renal Association (ERA) latest guidelines, published in 2021, suggest kidney transplantation is the optimal treatment for people with ESKD and a BMI up to 39.9 kg/m^2^, but conclude there is insufficient data to make a recommendation for patients with a BMI ≥40 kg/m^2^ [18]. The Kidney Disease: Improving Global Outcomes (KDIGO) guidelines suggest that transplantation in patients with BMI ≥40 kg/m^2^ should be approached with caution, and patients should be counselled on the increased post-operative risks [19]. British Transplantation Society guidelines state that although obesity is not an absolute contra-indication to transplantation, individuals with a BMI ≥40 kg/m^2^ are less likely to benefit [20].

Given this uncertainty within the clinical community, it is likely that many patients are denied transplantation on the basis of their BMI alone [21]. A recent survey of 23 transplant units in the UK showed that the overwhelming majority of units (20/23) had a BMI “cut-off”—by which patients who exceeded the BMI target were not considered for transplantation [22]. Others may be considered for transplantation upon reaching a target weight. The practice of delayed listing, however, may itself be harmful by increasing time spent on dialysis, thereby patients already at a higher risk for complications accrue further comorbidity [23, 24]. The dietary and lifestyle restrictions associated with ESKD, mean that achieving significant weight loss is particularly challenging for this cohort compared to the general population [25]. Latest guidelines support bariatric surgery for patients with BMI ≥40 kg/m^2^, or BMI ≥35 kg/m^2^ with additional co-morbidity, before transplantation [18]. However, access to timely bariatric surgery may be problematic in many regions.

It is accepted that transplantation confers a survival advantage for those patients with a BMI up to 39.9 kg/m^2^ [18]. However comparable literature on outcomes for patients with Class III obesity (BMI ≥40 kg/m^2^) is limited. We aimed to consider the graft and patient survival of recipients in the UK who had Class III obesity at the time of renal transplantation. Because national datasets are often unable to reliably capture relevant outcome measurements such as wound infections, biopsy-proven acute rejection, in-patient stay and other important metrics, we also analysed more granular short-, medium-, and long-term outcomes of the class III obese recipient cohort in the UK unit with the single greatest experience in this area.

## Materials and Methods

### Setting

The United Kingdom (UK) has a population of 67 million people, which is served by 23 adult renal transplant units. National Health Service Blood and Transplant (NHSBT) provides transplant services to the NHS across the UK. NHSBT is permitted to use patient identifiable information for service evaluation and safety monitoring without the consent of patients. Datasets are constructed based on information returned from individual transplant centres across the UK. The data available for analysis is anonymised at an individual level.

Northern Ireland (NI) is a distinct region within the UK with a population of 1.9 million people. All kidney transplants are performed in a single centre at the Regional Nephrology and Transplant Unit, Belfast City Hospital. Rates of obesity reflect those in the wider UK population [26], however, there is currently no bariatric surgery service available for patients in NI. Robot-assisted kidney transplantation is not utilised in NI. All transplant recipients are prospectively entered on the Northern Ireland Renal Transplant Database, which records patient characteristics and transplant outcomes. The Office of Research Ethics Committees Northern Ireland have given ethical approval for this database to be analysed to understand and improve renal services (Project IRAS ID 323151, REC Reference 23/NI/0034).

### Data Collection

#### UK National Data

Data on all adult patients receiving a kidney-only-transplant in the UK between April 2015–March 2022 inclusive were interrogated until the date of extraction (July 2022):

1. Recipient characteristics: BMI, age, sex, cause of renal failure, number of previous renal transplants, duration of prior renal replacement therapy (RRT).
2. Donor characteristics: age, sex, and type (living donor, deceased donor after brain or circulatory death).
3. Unit: number of recipients with class III obesity at time of transplant per adult renal transplant unit.
4. Outcomes: graft function (reported by treating clinicians as immediate, delayed (at least one dialysis session required in first post-operative week), and primary non-function) and survival time in days, and all-cause patient survival.

#### NI Regional Data

The outcomes of all patients who received a renal transplant with BMI ≥40 kg/m^2^ in NI between April 2015 and March 2022 were analysed until the date of extraction (February 2023). The median follow up time was 1740 days (range 483–2,930 days). Data were extracted from the prospectively collected NI Renal Transplant Database included, (in addition to the UK data):

5. Immunological details: HLA mismatch and the recipient’s calculated reaction frequency.
6. Transplant outcomes:
  a. Short term: organ ischaemic time, time to function (post-operative day of creatinine fall by at least 10%), dialysis requirement, critical care admissions, return to theatre, biopsy-proven acute rejection within 10 days, and length of stay in hospital for the index admission.
  b. Medium term: wound complications (infection requiring treatment with oral or intravenous antibiotics, requirement for tissue viability nursing (TVN) support, hernia), development of new-onset diabetes after transplantation (NODAT) and change in BMI at 1 year post-transplant.
  c. Long term: major adverse cardiac events (myocardial infarction, stroke, cardiac death, heart failure requiring hospitalisation, revascularisation).

### Statistics

In this study, parametric data were presented as mean ± standard deviation and non-parametric data as median and range. Analyses were performed on R v3.4.0 (R Foundation for Statistical Computing, Vienna, Austria). For national data, entries were checked for discrepancies between BMI at the time of listing and transplantation. For patients with a clear discrepancy between BMI at these two timepoints, we used height and weight to determine the accurate BMI. Erroneous entries were removed from the dataset before further analysis.

## Results

### UK National Data

During April 2015–March 2022, there were 22,845 adult kidney-only transplant operations in UK, of which just 44 (0.2%) were performed in individuals with a BMI ≥40 kg/m^2^ recorded at the time of transplantation. The median BMI was 46 kg/m^2^, range 40–49.4 kg/m^2^.

#### Donor Characteristics

Thirteen donors (29%) were living donors, 21 (48%) from donation after brain death and 10 (23%) from donation after circulatory death donors. Median donor age was 53 years (range 22–75 years). Donor characteristics are summarised in Table 1.

**TABLE 1:** Summary of donor characteristics.

UK National Data			
Age	Median: 53 years		
	Range: 22–75 years		
Type	Living Donor	DBD	DCD
	N = 13	N = 21	N = 10
	29%	48%	23%

NI Regional Data			
Age	Median: 52 years		
	Range: 22–58 years		
Sex	Male	Female	
	N = 11	N = 10	
	52%	48%	
Type	Living Donor	DBD	DCD
	N = 9	N = 5	N = 7
	43%	24%	33%

#### Recipient Characteristics

Twenty (45%) patients were male. The median age was 46 years (range 19–63 years). Only five (11%) patients were reported to have renal failure due to diabetic nephropathy, the prevalence of co-existent diabetes at the time of transplant is unknown. The primary renal disease was polycystic kidney disease in three patients (7%).

For most patients (*n* = 36, 82%) this was their first transplant. Five patients had one previous transplant, two had two previous transplants, one had three previous transplants. Six patients (14%) were transplanted pre-emptively, 35 (80%) were on dialysis at the time of transplant and RRT status at time of transplant was not recorded for three patients. Time on dialysis pre transplant ranged from 334–3,242 days (data available for 26/35 patients, median 1,232 days, mean 1,319 days). Recipient characteristics are summarised in Table 2.

**TABLE 2:** Summary of recipient characteristics.

UK National Data			
Age	Median: 46 years		
	Range: 19–63 years		
Sex	Male	Female	
	N = 20	N = 24	
	45%	55%	
RRT	Pre-emptive	Dialysis	Not recorded
	N = 6	N = 35	N = 3
	14%	80%	6%
Previous transplant	None	1	≥2
	N = 36	N = 5	N = 3
	82%	11%	7%

NI Regional Data			
Age	Median: 46 years		
	Range: 22–58 years		
Sex	Male	Female	
	N = 13	N = 8	
	62%	38%	
RRT	Pre-emptive	HD	PD
	N = 3	N = 14	N = 4
	14%	67%	19%
Previous transplant	None	1	≥2
	N = 19	N = 0	N = 2
	90%	0%	10%

#### Transplant Unit Details

**FIGURE 1:**
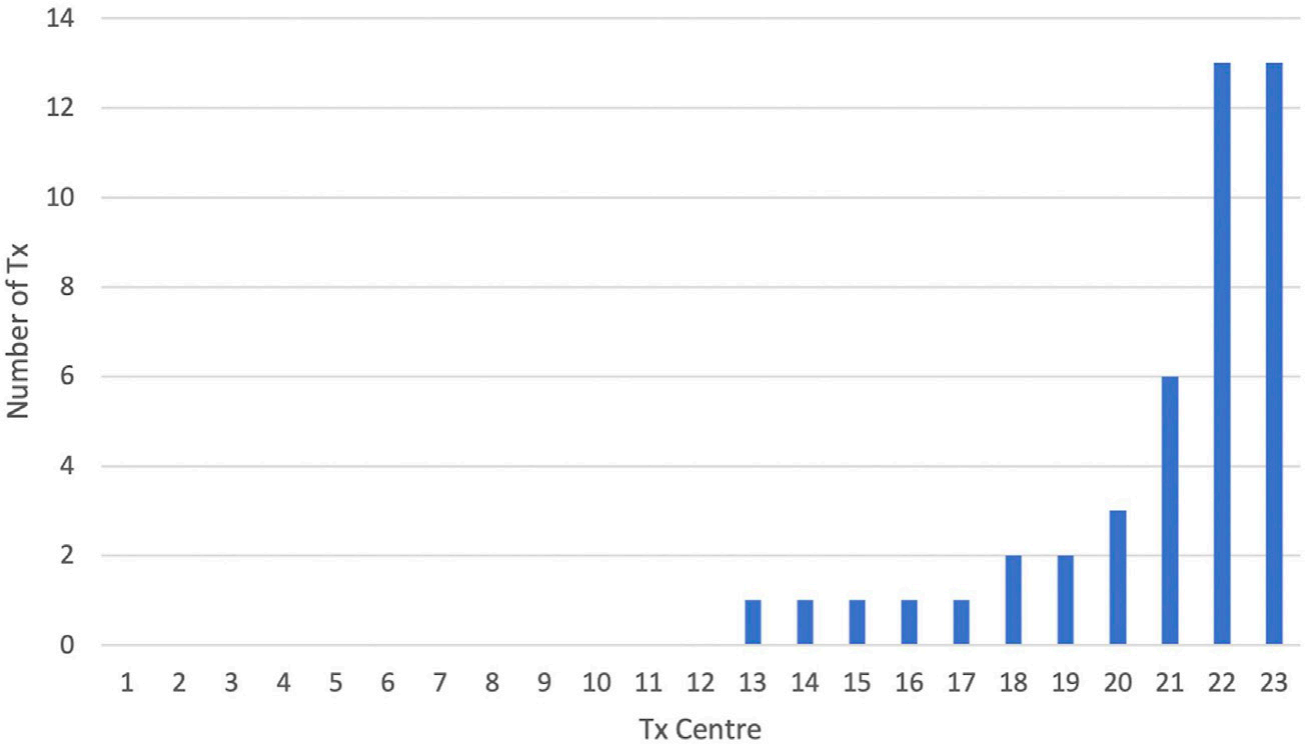
Number of patients with BMI ≥40 kg/m^2^ transplanted per UK transplant centre (*n* = 23) from 2015–2021.

Of the 23 adult renal transplant centres, the majority 12 (52%) did not transplant any patient with BMI ≥40 kg/m^2^ in this period, and four centres undertook this for a single patient only (Figure 1). Of the seven remaining centres, two were together responsible for transplanting 26 (59%) of the recipients with Class III obesity.

#### Survival Outcomes

Graft survival was recorded for 39 patients (median follow up 706 days, range 0–1,793 days). There was primary non-function in one, and death-censored transplant failure in two others (day 31 and day 226). The recorded graft survival was 36/39 (92%).

Patient survival is recorded for 34 patients (median follow up 710 days, range 24–1,793 days). Three deaths were recorded, at 24, 37, and 84 days post-transplant (90 days patient survival 31/34, 91%). Overall recorded patient survival is also 31/34 (91%).

### NI Regional Data

There were 841 adult renal transplants carried out in this region of which 21 (2.5%) were performed in 20 individuals with a BMI ≥40 kg/m^2^ at the time of transplantation. The mean BMI was 42 kg/m^2^, range 40–46 kg/m^2^. The median follow-up time is 57 months (range 15–96 months).

#### Donor Characteristics

Nine donors (43%) were living donors, 5 (24%) from donation after brain death and 7 (33%) from donation after circulatory death donors. Median donor age was 52 years (ranged 26–57 years).

#### Recipient Characteristics

Thirteen (62%) patients were male. The median age was 46 years (range 22–58 years). The most common cause (5, 25%) of renal failure was polycystic kidney disease (PKD). Only one patient had diabetic nephropathy, though 5 (25%) in total had diabetes at the time of transplant. One patient had a previous non-ST-elevation myocardial infarction (NSTEMI) with subsequent coronary stenting, and another was documented to have heart failure with preserved ejection fraction (estimated 55%).

Most transplants (19/21) were first transplants. One patient had three previous transplants, and one patient was transplanted twice during the study period. Three (14%) transplants were preemptive, 14 (67%) transplants were for patients on hemodialysis, and 4 transplants (19%) were for patients on peritoneal dialysis. The mean duration of renal-replacement therapy pre-transplant was 45 months (range 0–317 months). Recipient characteristics are summarised in Table 2.

#### Immunological Details

Most patients (15/20, 75%) were not previously sensitised. The calculated reaction frequency (cRF) ranged from 0%–97%. A-B-DR mismatches ranged from 1–6 (mean 3.4). Induction immunosuppression was 500 mg of intravenous methylprednisolone intra-operatively, and Basiliximab 20 mg pre-operatively and on day 4 post-transplant for younger patients (≤40 years) or if there was a poorer HLA match (2DR, or 2B & 1 DR). Standard oral immunosuppression was Prednisolone 20 mg OD, Mycophenolate Mofetil 1g BD, and Tacrolimus with a trough level typically 12 ± 2 μg/L.

#### Short-Term Outcomes

**FIGURE 2:**
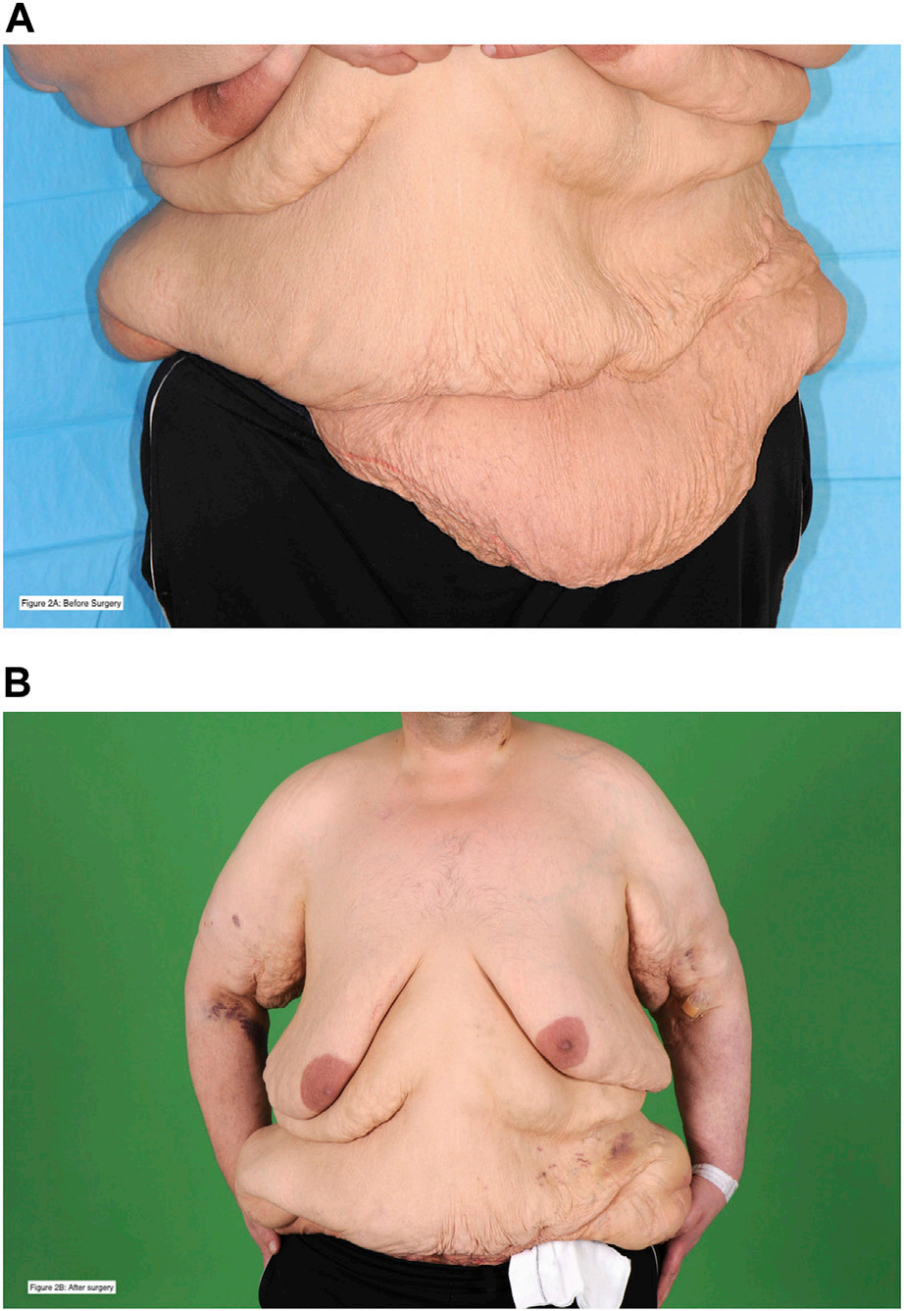
This is a 38 year-old patient who was on haemodialysis at the time of transplant. He had a previous kidney transplant in the right iliac fossa that failed due to graft thrombosis. He developed an incisional hernia, underwent hernia repair, and after further weight loss was left with abdominal wall asymmetry and excess skin. He underwent combined renal transplantation in the left iliac fossa with apronectomy and abdominoplasty. Picture **(A)**, with a blue background, is before surgery. Picture **(B)**, with a green background, is post-surgery. The patient is alive with a functioning graft 4 years following transplantation. The patient has provided written and verbal consent for these photographs to be taken and shared for these purposes.

A single patient required admission to critical care. The first admission was unplanned (major post-operative haemorrhage with subsequent graft loss), and the second admission (following a subsequent transplant with combined apronectomy and abdominoplasty, Figure 2) was planned. No other patient in the cohort required admission to critical care during this period.

Nine (43%) patients required dialysis following transplantation, in 8 (including 3/9 from living donors) this was due to delayed graft function. One patient required dialysis due to primary graft failure. The median time to a 10% fall in creatinine was 5 days (range 1–56 days). Five patients (24%) developed biopsy proven acute cellular rejection (ACR) within 10 days of transplantation. All were managed successfully with intravenous methylprednisolone and up-titration of oral immunosuppression. There were no episodes of antibody-mediated rejection.

The median length of stay for the index admission was 9.0 days (range: 4–21 days). There was no significant difference between length of stay for deceased or living donor recipients.

#### Medium-Term Outcomes

Fourteen patients had wound related problems post-operatively. Five patients (24%) developed incisional hernia and five patients (24%) developed wound infection. There was impaired wound healing requiring specialist input in 6 patients (28%).

Five patients had diabetes at the time of transplant. Of the remaining patients 5 (31%) developed NODAT during the follow-up period. For those who did not have diabetes at the time of transplant, the median HbA1c pre-transplant was 31.5 mmol/mol (range 27–47 mmol/mol), and it was 36 mmol/mol at 1 year post-transplant (range 21–67 mmol/mol).

BMI at 1 year post-transplant was available for 16 of the 17 patients alive with a functioning graft. The median percentage-change in BMI was −2.7%, representing an overall trend of weight loss amongst patients in the cohort, though this ranged from −26% to +22%. The percentage-change in BMI at 3 years was available for all 9 patients alive with a functioning graft. Median percentage change in BMI at 3 years was −0.5%, with a range from −21% to +26%.

#### Long-Term Outcomes

**FIGURE 3:**
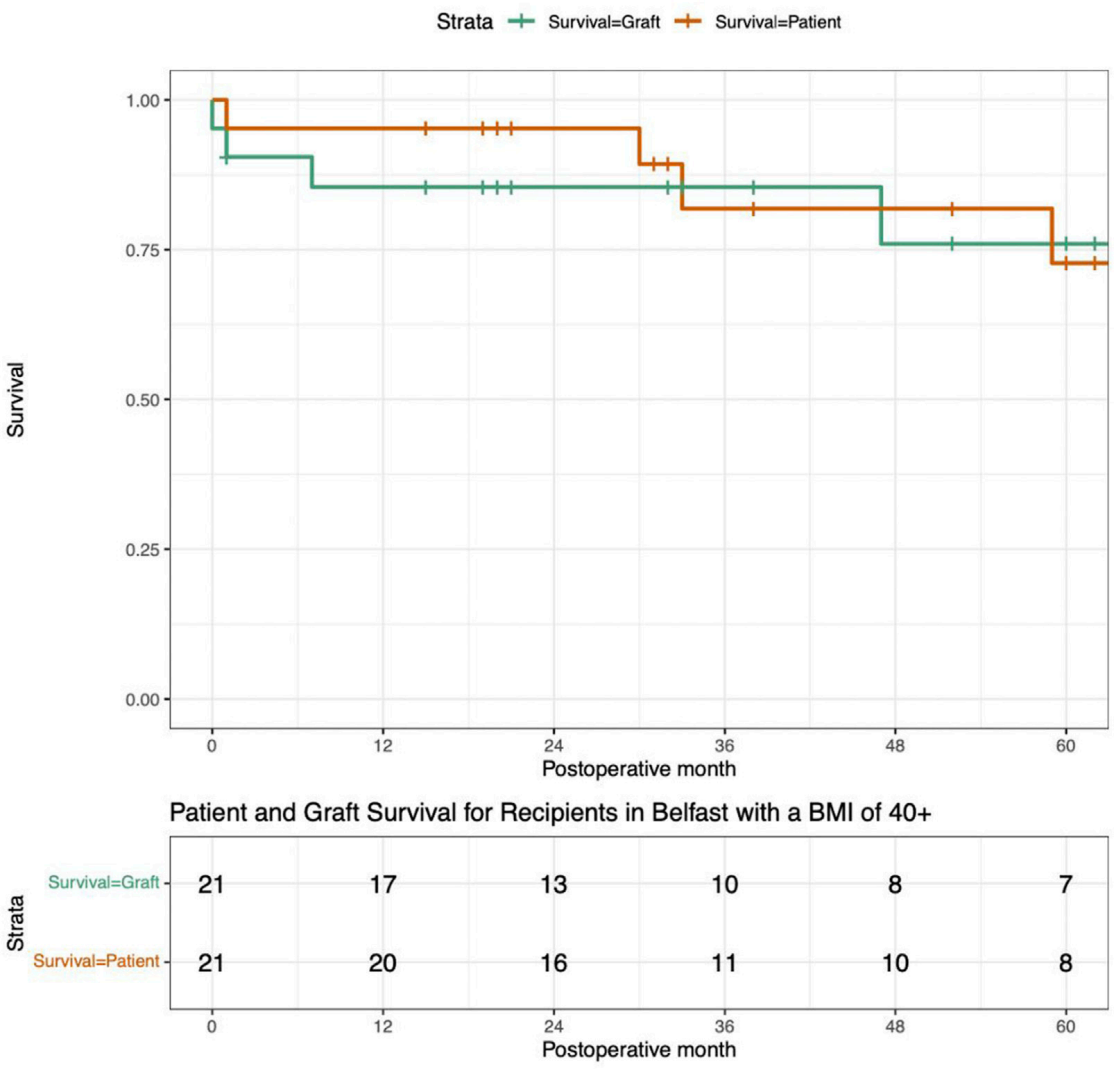
These Survival Curves present the patient survival and death censored graft survival of this cohort of patients who underwent renal transplantation with a BMI ≥40 kg/m^2^.

One-year follow-up was available for all patients. Patient and death-censored graft survival was 95% and 85% at 1 year post-transplant. Three-year outcomes were available for 14 patients, patient and death censored graft survival was 79% and 82% respectively. Of 9 patients transplanted at least 5 years ago, 8 are still alive, and 7 have a functioning transplant (77% graft survival). Survival curves are presented in Figure 3.

The reasons for graft loss were varied: early failure secondary to graft thrombosis, non-recovery following recurrent acute kidney injury within a few weeks, late acute rejection secondary to non-compliance during COVID-19 pandemic, and acute cortical necrosis due to life-threatening ischaemic bowel.

In total, four patients died following transplantation. One patient died due to respiratory failure following COVID-19 infection, one patient died due to metastatic pancreatic cancer, one patient died due to bowel ischaemia, and one patient died of a presumed cardiac event. Amongst the remaining patients, there were no episodes of stroke, myocardial infarction, hospitalisation with heart failure, or requirement for coronary revascularisation following transplantation.

## Discussion

This study provides much needed evidence on the outcomes of kidney transplantation in patients with a BMI ≥40 kg/m^2^. A particular strength of this study is our presentation of registry data alongside individual patient data from the highest-volume UK centre for transplants of this type, enabling a more detailed analysis across a range of parameters. The current evidence base for transplantation in individuals with Class III obesity does not allow for strong recommendations to be made [18]. This is partly due to the limited number of centres who perform transplantation at extremes of weight. Lack of support by national and international guidelines may perpetuate this reluctance, and subsequent paucity of data.

### UK Transplant Practice

Two large registry studies have shown an overall mortality benefit of transplantation irrespective of BMI [17, 27]. with an appreciation that transplanting such individuals is generally associated with good outcomes, although with increased morbidity and mortality compared to the non-obese recipient.

However, despite this, our review of national activity in transplantation for patients reported to have a BMI ≥40 kg/m^2^, demonstrates no appreciable increase in transplantation rates for this patient cohort in the UK. From 2004–2010, 38 patients with a BMI ≥40 kg/m^2^ were transplanted in the UK. In 2015–2021, 44 patients in this BMI category were transplanted. This static position in transplant numbers exists despite the substantial rise in the prevalence of obesity, including in those with ESRD, and the increase in renal transplant numbers overall in this period.

The detail of this study reveals that a reluctance to perform transplantation in this group of patients pervades the majority of UK transplant units. Only a quarter (6/23) of centres transplanted more than one patient with BMI ≥40 kg/m^2^, and half (12/23) did not perform a single transplant for patients within this BMI category in this 7 years period. Just two units undertook the majority of recorded transplants (26/44). This corresponds to previous work, which showed that the majority centres in the UK operationalised “BMI cut-offs” [22].

### Transplant Complexities

In our centre, the proportion of patients with Polycystic Kidney Disease (PKD) represented in the study is higher than expected (25% of patients with BMI ≥40 kg/m^2^, compared to 15% of the total cohort of transplant patients in our centre in the study period). Large polycystic kidneys may contribute to some of the excess body weight for this cohort [28], and policies which operate BMI cut-offs may disproportionately disadvantage patients with PKD. Diabetic nephropathy, which may be anticipated to be more common in a cohort of patients with marked obesity, was the cause of ESKD in a single patient. Undoubtedly this reflects the careful selection in our Unit of the patients with class III obesity that proceed to transplant, with a nuanced and individualised consideration of the constellation of comorbidities for each. This is reflected in the age at transplantation, with the oldest recorded in our region being 58 years and nationally 63 years.

Critical in the selection of candidates with class III obesity for transplantation is consideration of the likelihood of complications and the physiological reserve to deal with a potential stormy post-transplant course. The granularity of the regional data allowed the nature and rate of complications to be detailed.

Delayed graft function is likely. The rate of delayed graft function is particularly unusual in those patients in receipt of a living donor transplant. In our centre in this period only 9% of living donor transplants did not function immediately, compared to 33% in this cohort (unpublished data). The rates of ACR are also higher: 7% of our patients overall compared to 24% in patients with BMI ≥40 kg/m^2^ (unpublished data). It is important, however, to highlight that this can be successfully managed without deviation from normal protocol. The median length of stay was longer than typical for transplantation in our unit in this period (approximately 9 rather than 6 days), though not excessive. Within our practice the utilization of critical care is low and can be successfully anticipated for certain patients.

As expected, wound issues are common, though not inevitable (a third did not have any issue). This may require additional antibiotic therapy, input from a specialised Tissue Viability Team, and in certain cases, further operative treatment (e.g., hernia repair). NODAT developed in a substantial number, but not all patients, highlighting the need for regular monitoring and a multidisciplinary approach to post-transplant care.

Mortality is higher than our local and published national outcomes [31]. The 1 year patient survival (95%) is lower than the 98% for deceased donor and 99% for living donor kidney transplant recipients. There is an even greater difference in 1 year graft survival: 85% is considerably lower than our overall cohort, (92% in deceased donor and 99% in living donor transplantation). Interestingly only one patient had a (presumed) major adverse cardiac event, and the recorded deaths were due to disparate causes, as may be anticipated in a group with class III obesity.

It is important to interpret the outcomes for this group with comparison to the expected mortality of living with obesity and CKD. The survival benefit of transplantation should be compared to the next best alternative, accounting for the potential difficulty in achieving adequate dialysis for patients with BMI ≥40 kg/m^2^, particularly in the time constraints of in-centre haemodialysis.

### Mitigation of Risk

Obviously, weight loss before transplantation is the one certain way to reduce the risks associated with renal transplantation and minimise subsequent complications. Achieving and sustaining weight loss is challenging even for those without renal failure and significant weight loss is unlikely for most patients with ESKD without surgical intervention. Recently published guidelines suggest that transplant candidates with BMI ≥40 kg/m^2^ are considered for bariatric surgery before transplantation, with the intention of successful weight loss (to reduce BMI ≤39.9 kg/m^2^) [18]. Bariatric surgery itself, however, is not without associated risk [31–33]. The majority of patients in our centre have ultimately had a successful transplant outcome, and if selection criteria can be further refined to identify such individuals, it could be argued that the risks of bariatric surgery, particularly combined with the increased wait-time to transplantation, may outweigh the risk of transplantation alone with BMI ≥40 kg/m^2^. The evolution of new medications for the treatment of obesity, such as liraglutide, may change the risk vs. benefit profile of weight loss interventions pre-transplantation but their efficacy in patients with ESKD and Class III obesity remains uncertain [34].

If proceeding with transplantation in patients persistently with BMI ≥40 kg/m^2^, then the risk of a poor outcome could be reduced by an elective operation with a living donor transplant. In our centre this was the setting for almost half of the transplant procedures for those with class III obesity. This is reflective of our transplant practice overall but is in contrast to the national UK practice. Within the national cohort, there were just 13 living donor transplants in this cohort over the 7 years period. Yet the planned nature of this work affords the opportunity to optimise the patient’s peri-operative status and arrange for experienced surgical and anaesthetic teams, in addition to preparing for critical care use for the small minority of patients who may require it. In these patients where the operative and peri-operative risk high, the benefits of living donation will exceed even the standard benefits of such transplants for patients with normal BMI. This ability to reduce some of the potentially avoidable risk may create a more favourable risk:benefit ratio in individualised decision making. Robot-assisted kidney transplantation has been reported to decrease wound morbidity in obese patients but practice is not yet widespread, and it is not available within our region [29, 30].

As with all clinical practice, experiential learning is of critical importance. For units with limited experience, embarking on the occasional transplant in a patient with a BMI ≥40 kg/m^2^, is daunting and provides little opportunity to minimise the risks. A limited number of higher-volume centres are likely to provide a safer service model for this cohort of patients. It may be beneficial to have clear referral pathways to centres that will consider transplantation for patients where BMI alone is a precluding factor in their local unit, and thereby minimising inequity of access to transplantation.

### Limitations

We are aware that BMI is an imperfect measure. Whilst this is a limitation, it reflects the data most likely to be available to clinicians at the time of assessment and transplant listing. Future work could look at the acceptability and feasibility of obtaining surrogate measures, such as waist circumference and waist to hip ratio, at clinic visits [18]. Furthermore, the fat distribution is likely to be relevant to outcomes: experientially central male adiposity is associated with greater complications than a female with relatively more adiposity in hips and thighs. The impact of this has not be described in terms of transplant outcomes.

A second limitation of this study is that we have only analysed the outcomes of patients who have been transplanted. Comparing outcomes to patients with lower or normal BMIs following transplantation is not useful, as the results for obese patients will inevitably be worse. It would be of interest to quantify the outcomes for patients with Class III obesity who are not transplanted. The most suitable comparator group may be those individuals who remain listed with BMI ≥40 kg/m^2^. However, given the demonstrated reluctance, at least within the UK, to transplant such individuals, the comparator group is small, and would not include those otherwise suitable for transplantation who are not given the opportunity to be listed for transplantation.

A final limitation is that of registry data. Our analysis was limited by the amount of missing (or erroneous) data recorded in the National UK Transplant Registry. As has been reported in other studies, this restricts the potential usefulness of conclusions, particularly when analysing data for a very small number of patients [17]. It is notable that our centre, with reliable accuracy of data collection, had more patients transplanted with class III obesity than were recorded in the national statistics.

### Future Studies

Further research may take the form of a prospective study, recording a variety of metrics of obesity, with long-term follow up from the point of initial assessment. It would also be of interest to understand how transplant nephrologists and surgeons make the complex decisions to list individuals with BMI ≥40 kg/m^2^ for transplantation. Not all patients with a BMI ≥40 kg/m^2^ were listed during the study period. We have presented the outcomes of a cohort of patients who had ESKD and BMI ≥40 kg/m^2^ but whose other comorbidities and functional status, in combination with their BMI, meant they were deemed acceptable for transplantation. This sophisticated approach to listing, which assesses an individual’s overall risk profile, rather than a single factor is likely to increase access to transplantation for all those who may benefit.

## Conclusion

Renal transplantation is a lifesaving and life-changing intervention. Arbitrary cut-off values for BMI artificially restrict access to the waiting list and may exclude patients who could otherwise benefit from transplantation. This study shows that favourable outcomes for patients who undergo renal transplantation with BMI ≥40 kg/m^2^ but that despite this, few centres in the UK offer this therapeutic option to their patients. Rather than a “BMI cut-off,” patients will benefit most from an individualised approach to risk stratification; accounting for their BMI, other co-morbidities, the potential benefits of pre-emptive transplantation, and the adverse consequences of remaining on maintenance dialysis therapy. National consideration of concentrating expertise in this group of recipients in a smaller number of higher volume transplant centres may be useful.

## Data Availability

The raw data supporting the conclusion of this article will be made available by the authors, without undue reservation.
